# Supplementary material for: Transitory impact of subclinical Shigella infections on biomarkers of environmental enteropathy in children under 2 years
Source: PLoS Negl Trop Dis. 2025 May 29;19(5):e0012791. doi: 10.1371/journal.pntd.0012791 (PMC12143526; doi:10.1371/journal.pntd.0012791)
Supplement: S1 Table — (PDF) [file pntd.0012791.s001.pdf]

S1 Table. Monthly EE biomarker concentration associations from time of index subclinical *Shigella* detection through 6-months post-detection.

Month after index *Shigella* detection	MPO concentration difference ln[ng/mL] (95% CI)	NEO concentration difference ln[nmol/L] (95% CI)	AAT concentration difference ln[mg/g] (95% CI)
0	0.30 (0.23, 0.37)	0.01 (-0.05, 0.07)	-0.02 (-0.08, 0.04)
1	-0.04 (-0.17, 0.08)	-0.18 (-0.28, -0.07)	-0.04 (-0.15, 0.07)
2	0.06 (-0.08, 0.20)	-0.13 (-0.25, -0.01)	-0.09 (-0.22, 0.03)
3	-0.04 (-0.13, 0.06)	-0.14 (-0.22, -0.05)	-0.08 (-0.16, 0.01)
4	-0.08 (-0.23, 0.07)	-0.07 (-0.20, 0.07)	-0.08 (-0.20, 0.05)
5	-0.18 (-0.35, -0.00)	-0.14 (-0.30, 0.01)	0.02 (-0.12, 0.17)
6	-0.16 (-0.26, -0.04)	-0.13 (-0.24, -0.02)	-0.13 (-0.24, -0.03)

MPO: myeloperoxidase; NEO: neopterin; AAT: alpha-1-antitrypsin
