# Supplementary material for: Transitory impact of subclinical Shigella infections on biomarkers of environmental enteropathy in children under 2 years
Source: PLoS Negl Trop Dis. 2025 May 29;19(5):e0012791. doi: 10.1371/journal.pntd.0012791 (PMC12143526; doi:10.1371/journal.pntd.0012791)
Supplement: S2 Table — (PDF) [file pntd.0012791.s002.pdf]

S2 Table. Biomarker concentrations among *Shigella* infections and *Shigella* infections with coinfections

	***Shigella*-only Infections** (N=120)	***Shigella* Infections with Coinfections** (N=3,385)	***Shigella* Infection Total** (N=3,505)
MPO ln(ng/mL), Median [Q1, Q3]	7.97 [7.07, 9.21]	8.53 [7.67, 9.41]	8.53 [7.66, 9.40]
NEO ln(nmol/L), Median [Q1, Q3]	7.50 [6.36, 8.12]	7.06 [6.12, 7.86]	7.07 [6.13, 7.87]
AAT ln(mg/g), Median [Q1, Q3]	-1.49 [-2.46, -0.70]	-1.32 [-2.06, -0.66]	-1.33 [-2.06, -0.66]

MPO: myeloperoxidase; NEO: neopterin; AAT: alpha-1-antitrypsin
