# Supplementary material for: Transitory impact of subclinical Shigella infections on biomarkers of environmental enteropathy in children under 2 years
Source: PLoS Negl Trop Dis. 2025 May 29;19(5):e0012791. doi: 10.1371/journal.pntd.0012791 (PMC12143526; doi:10.1371/journal.pntd.0012791)
Supplement: S3 Table — (PDF) [file pntd.0012791.s003.pdf]

S3 Table. Biomarker concentrations among the 8 MAL-ED study sites

Study Sites	Study Size (N = 34,654) N (%)	MPO ln(ng/mL) Median [Q1, Q3]	NEO ln(nmol/L) Median [Q1, Q3]	AAT ln(mg/g) Median [Q1, Q3]
Bangladesh	4,318 (12.5%)	8.24 [7.55, 9.05]	7.15 [6.09, 7.91]	-0.90 [-1.55, -0.26]
Brazil	2,839 (8.2%)	7.88 [6.84, 8.92]	7.30 [6.70, 7.94]	-1.19 [-1.78, -0.63]
India	4,768 (13.8%)	8.95 [7.99, 9.92]	7.54 [6.83, 8.14]	-1.11 [-1.72, -0.46]
Nepal	5,045 (14.6%)	8.46 [7.63, 9.31]	7.50 [6.96, 7.95]	-0.78 [-1.41, -0.17]
Peru	4,212 (12.2%)	9.00 [8.07, 9.88]	7.84 [7.26, 8.37]	-0.81 [-1.53, -0.21]
Pakistan	4,641 (13.4%)	8.38 [7.34, 9.34]	6.26 [5.57, 6.94]	-1.80 [-2.59, -0.96]
South Africa	4,578 (13.2%)	8.49 [7.67, 9.30]	8.22 [7.64, 8.85]	-1.30 [-2.10, -0.62]
Tanzania	4,253 (12.3%)	8.60 [7.68, 9.54]	6.76 [5.79, 7.57]	-1.33 [-2.07, -0.61]
