# Supplementary material for: Transitory impact of subclinical Shigella infections on biomarkers of environmental enteropathy in children under 2 years
Source: PLoS Negl Trop Dis. 2025 May 29;19(5):e0012791. doi: 10.1371/journal.pntd.0012791 (PMC12143526; doi:10.1371/journal.pntd.0012791)
Supplement: S1 Fig — Each plot shows EE biomarker natural log concentration differences and 95% confidence intervals comparing non-diarrheal stool samples with and without Shigella detection at month 0. Concentrations were measured monthly from the index Shigella-positive detection through 6-months post-detection. (PDF) [file pntd.0012791.s004.pdf]

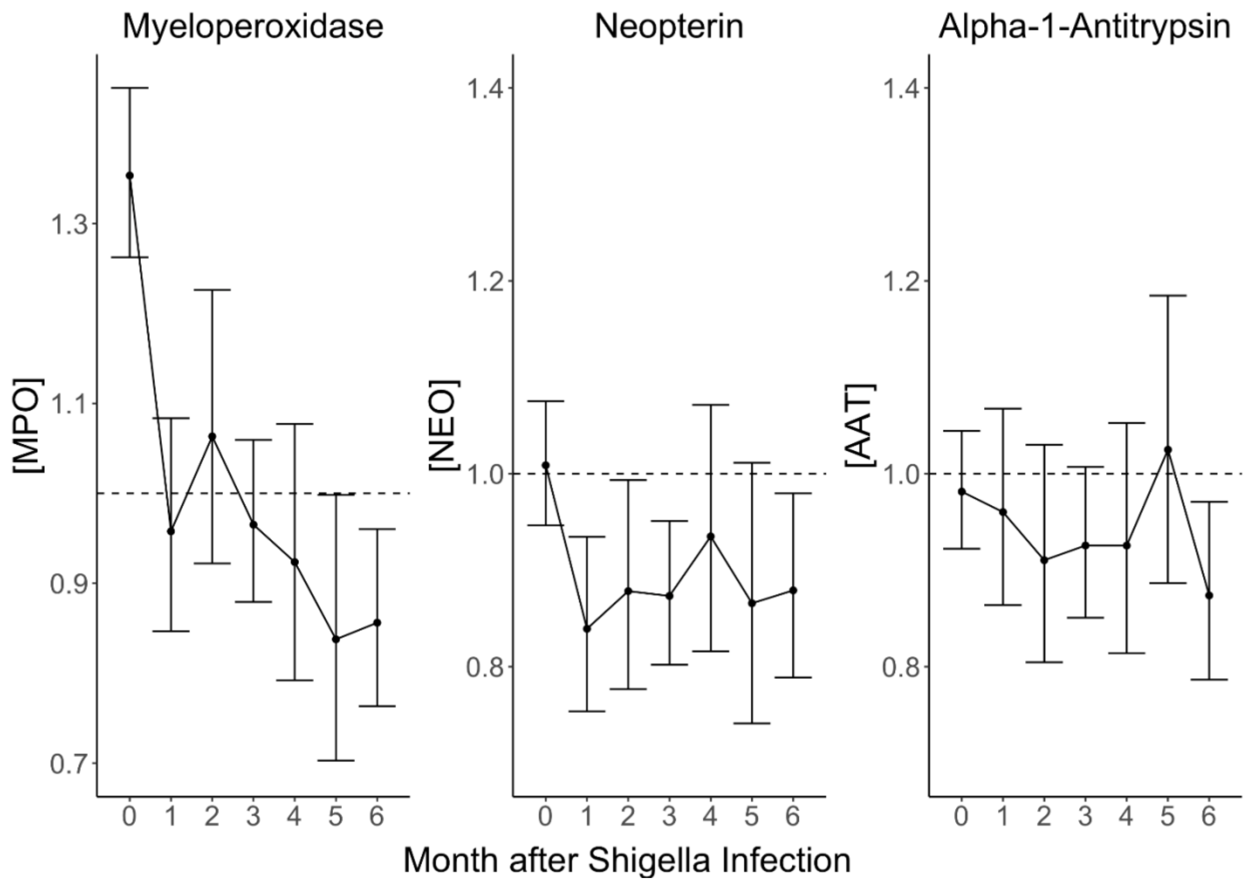

S1 Fig. Association of *Shigella* infections with raw EE biomarker concentrations values over time.

Each plot shows EE biomarker natural log concentration differences and 95% confidence intervals comparing non-diarrheal stool samples with and without *Shigella* detection at month 0. Concentrations were measured monthly from the index *Shigella*-positive detection through 6-months post-detection.
