# Supplementary material for: Transitory impact of subclinical Shigella infections on biomarkers of environmental enteropathy in children under 2 years
Source: PLoS Negl Trop Dis. 2025 May 29;19(5):e0012791. doi: 10.1371/journal.pntd.0012791 (PMC12143526; doi:10.1371/journal.pntd.0012791)
Supplement: S2 Fig — Each plot shows EE biomarker natural log concentration differences and 95% confidence intervals comparing non-diarrheal stool samples with and without Shigella detection at month 0. The dashed black line represents the modeling while adjusting for age which completely overlaps with the dashed orange line that represents the model adjusting for age and detrending for age. The green line is the model only detrending for age. The blue line is the model adjusting for age and other infections at the time of the biomarker measurement (i.e., subsequent infections). The purple line is the model adjusting for age, other infections at the time of the biomarker measurement (i.e., subsequent infections), and Shigella infections at the time of the biomarker measurement (i.e., subsequent Shigella infections). (PDF) [file pntd.0012791.s005.pdf]

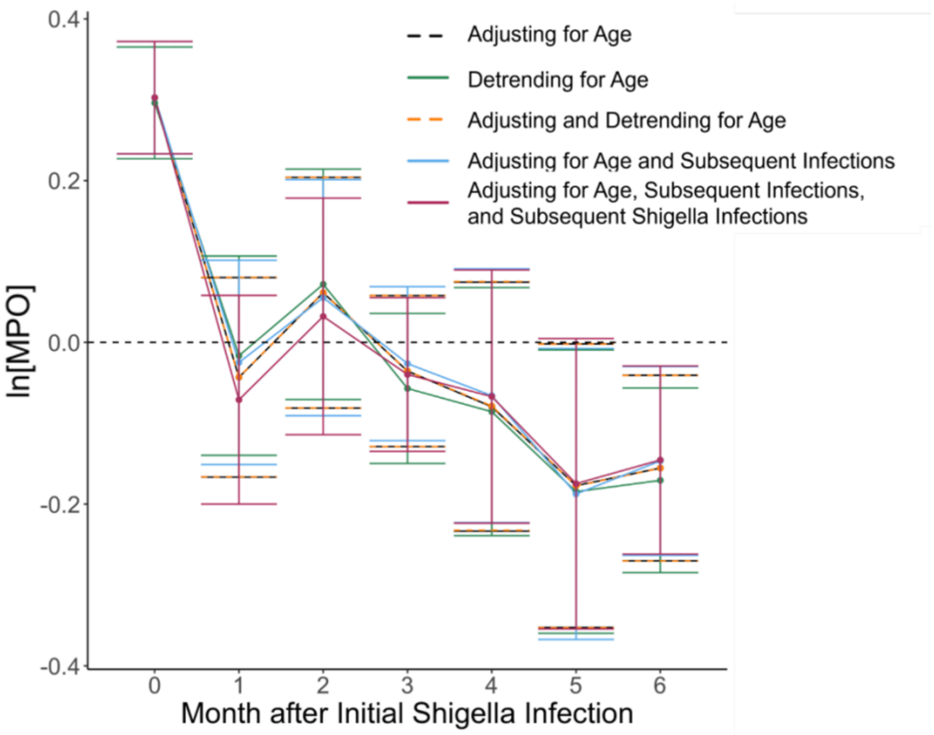

S2 Fig. Longitudinal impact of *Shigella* infections on MPO concentrations by varying specifications of age and adjustment for subsequent infections. Each plot shows EE biomarker natural log concentration differences and 95% confidence intervals comparing non-diarrheal stool samples with and without *Shigella* detection at month 0. The dashed black line represents the modeling while adjusting for age which completely overlaps with the dashed orange line that represents the model adjusting for age and detrending for age. The green line is the model only detrending for age. The blue line is the model adjusting for age and other infections at the time of the biomarker measurement (i.e., subsequent infections). The purple line is the model adjusting for age, other infections at the time of the biomarker measurement (i.e., subsequent infections), and *Shigella* infections at the time of the biomarker measurement (i.e., subsequent *Shigella* infections).
