# Supplementary material for: Transitory impact of subclinical Shigella infections on biomarkers of environmental enteropathy in children under 2 years
Source: PLoS Negl Trop Dis. 2025 May 29;19(5):e0012791. doi: 10.1371/journal.pntd.0012791 (PMC12143526; doi:10.1371/journal.pntd.0012791)
Supplement: S3 Fig — Each plot shows EE biomarker natural log concentration differences and 95% confidence intervals comparing non-diarrheal stool samples with and without Shigella detection at month 0. The association of subclinical Shigella infection on fecal biomarkers among children younger the age of 24 months is represented by the black line. The association of subclinical Shigella infection on fecal biomarkers among children younger than the age of 18 months is represented by the dashed blue line. (PDF) [file pntd.0012791.s006.pdf]

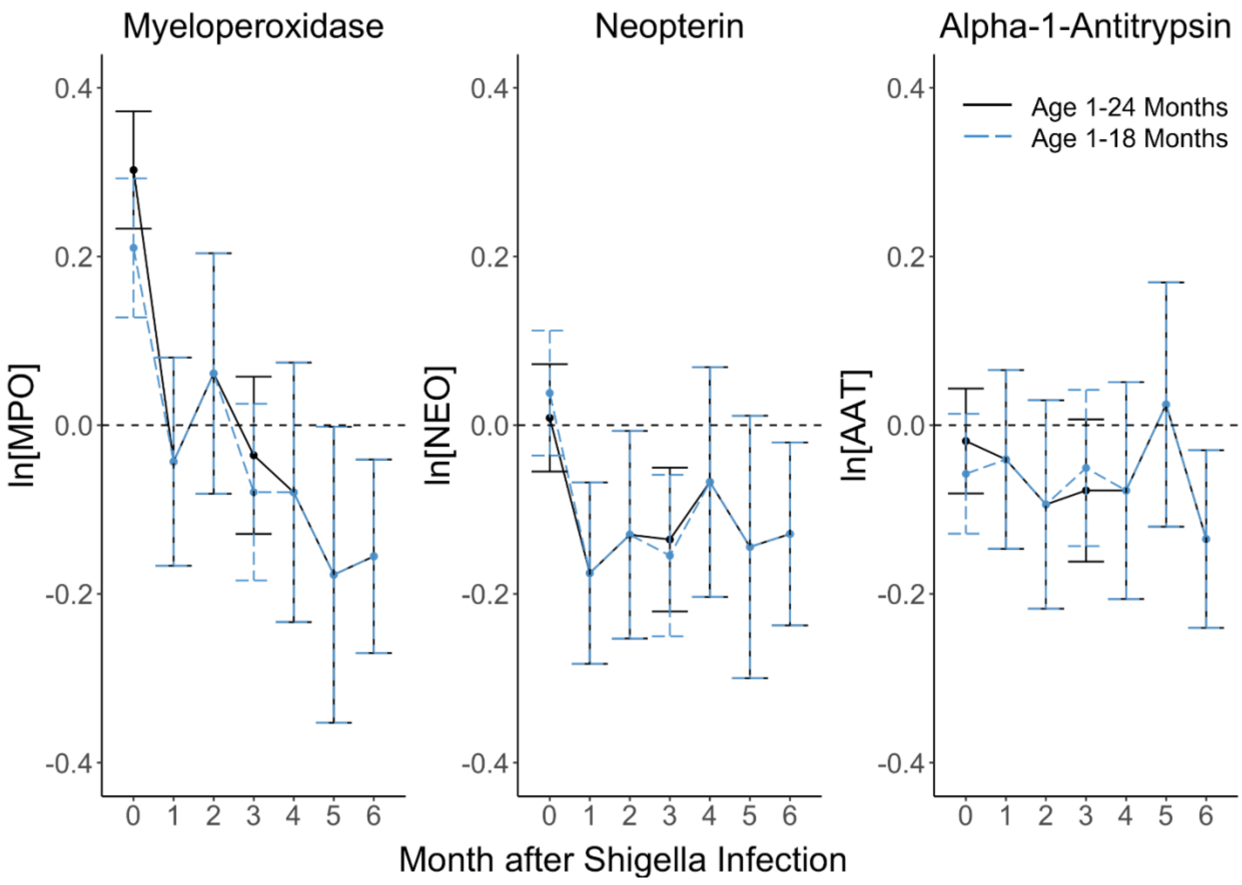

S3 Fig. Longitudinal impact of *Shigella* infections on biomarker concentrations among children 1-24 months of age and among children 1-18 months of age. Each plot shows EE biomarker natural log concentration differences and 95% confidence intervals comparing non-diarrheal stool samples with and without *Shigella* detection at month 0. The association of subclinical *Shigella* infection on fecal biomarkers among children younger the age of 24 months is represented by the black line. The association of subclinical *Shigella* infection on fecal biomarkers among children younger than the age of 18 months is represented by the dashed blue line.
