# Supplementary material for: Transitory impact of subclinical Shigella infections on biomarkers of environmental enteropathy in children under 2 years
Source: PLoS Negl Trop Dis. 2025 May 29;19(5):e0012791. doi: 10.1371/journal.pntd.0012791 (PMC12143526; doi:10.1371/journal.pntd.0012791)
Supplement: S4 Fig — Each plot shows EE biomarker natural log concentration differences and 95% confidence intervals comparing non-diarrheal stool samples with and without Shigella detection at month 0. The association of subclinical Shigella infection on fecal biomarkers among children with any co-infection is represented by the black line. The association of subclinical Shigella infection on fecal biomarkers among children younger with viral only co-infections (no bacterial or parasitic co-infections) is represented by the dashed green line. (PDF) [file pntd.0012791.s007.pdf]

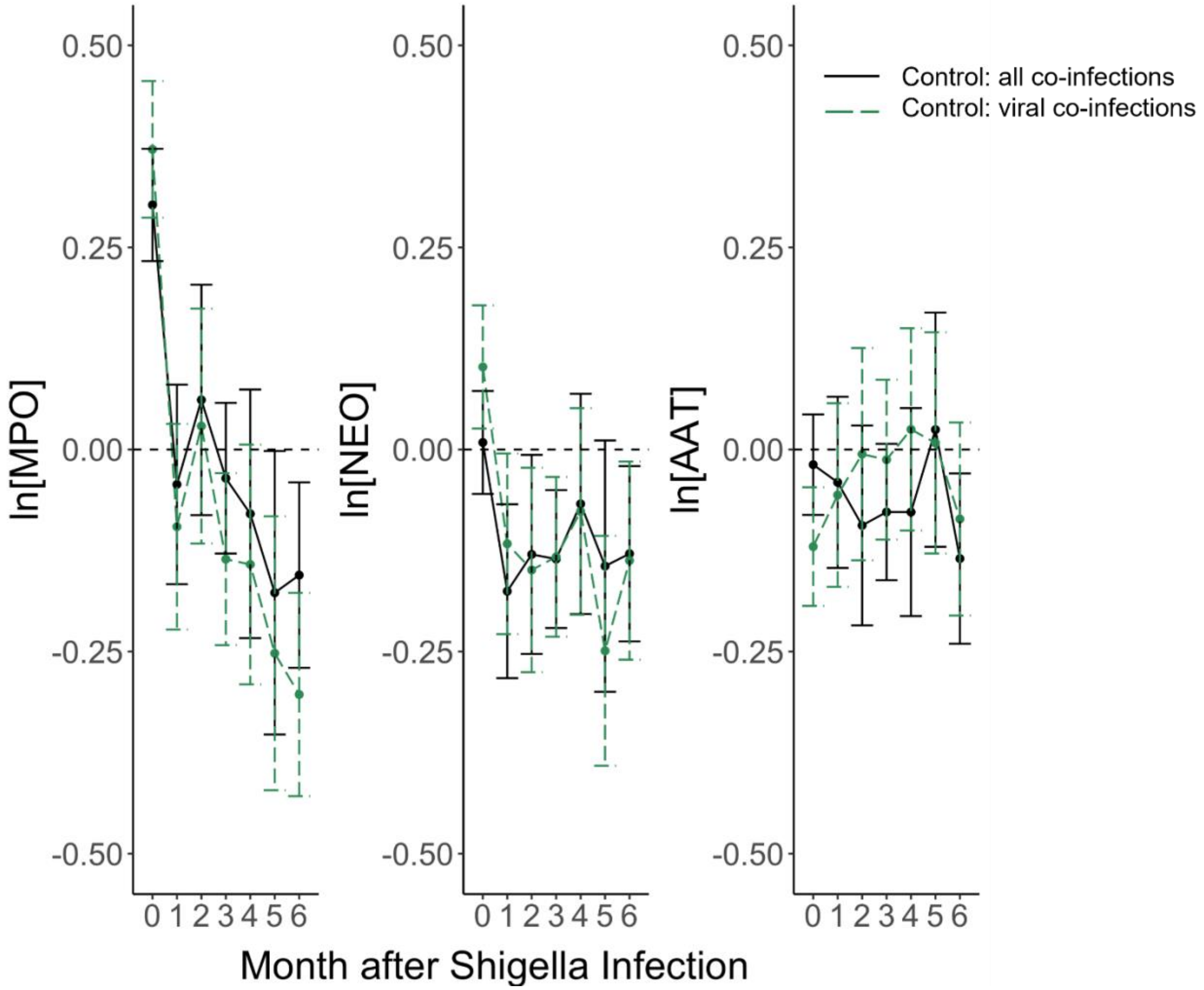

S4 Fig. Longitudinal impact of *Shigella* infections on biomarker concentrations compared with a control group consisting of stools with no *Shigella* infections and co-infections and compared with a control group consisting of no *Shigella* infections and only viral co-infections. Each plot shows EE biomarker natural log concentration differences and 95% confidence intervals comparing non-diarrheal stool samples with and without *Shigella* detection at month 0. The association of subclinical *Shigella* infection on fecal biomarkers among children with any co-infection is represented by the black line. The association of subclinical *Shigella* infection on fecal biomarkers among children younger with viral only co-infections (no bacterial or parasitic co-infections) is represented by the dashed green line.
